# Generation and Characterization of a Sexual Tetraploid Genotype of Seashore Paspalum (*Paspalum vaginatum*) for Novel Breeding Strategies

**DOI:** 10.3390/plants15182758

**Published:** 2026-09-09

**Authors:** Patricia Elda Novo, Ana María Gonzalez, Matías Oscar Espindola, Norma Estefanía Gómez, Florencia Galdeano, Carlos Alberto Acuña

**Affiliations:** Instituto de Botánica del Nordeste, Consejo Nacional de Investigaciones Científicas y Técnicas, Facultad de Ciencias Agrarias, Universidad Nacional del Nordeste, Corrientes 3400, Corrientes, Argentina; pnovo@agr.unne.edu.ar (P.E.N.); anagonzalez.ibone@gmail.com (A.M.G.); matiase68@gmail.com (M.O.E.); normaestefania21@gmail.com (N.E.G.); floppy@agr.unne.edu.ar (F.G.)

**Keywords:** *Paspalum*, sexual tetraploid, chromosome doubling, polyploidization, reproductive mode, self-incompatibility, turfgrass breeding

## Abstract

Breeding *Paspalum vaginatum* has been limited to the diploid germplasm; however there is a rich diversity in closely related species that may be utilized. The species is diploid and outcrossing due to self-incompatibility. This research aimed to generate a chromosome-doubled tetraploid and evaluate the effects of chromosome doubling on phenotype and reproductive mode. Both seeds and seedlings from a diploid population were treated with 0.2% colchicine for either 24 or 48 h. Morphological, anatomical, and reproductive traits were evaluated in both cytotypes. Megasporogenesis and megagametogenesis were assessed using complementary histological and optical approaches, and seed set was evaluated under self- and open-pollination. A tetraploid genotype was successfully generated from seedlings treated with colchicine for 24 h with an efficiency of 0.2%. The size of tillers, leaves, inflorescences, stomata, and pollen grains increased in the tetraploid, which also exhibited darker green coloration and greater vigor. Embryo sac development was similar in both cytotypes, indicating that the induced tetraploid remained sexual. No seed set was observed under self-pollination, whereas limited seed set under open-pollination confirmed that the tetraploid is an outcrosser. This new tetraploid provides a valuable bridge parent for introgressing genetic variation from cross-compatible polyploid *Paspalum* species through interspecific hybridization and the manipulation of apomixis.

## 1. Introduction

*Paspalum vaginatum* Sw. (seashore paspalum) is a warm-season perennial grass that is particularly well adapted to moist and salt-affected environments commonly found in coastal regions [1]. It is used for forage, ground cover, and, most importantly, turf in tropical and subtropical areas [2]. The combination of desirable turf characteristics and tolerance to a range of environmental stresses has promoted the widespread use of seashore paspalum in sports turf systems. It has become a popular choice for golf courses worldwide, especially in coastal regions and areas near brackish or highly saline water sources [1]. Its suitability for establishment under harsh environmental conditions is illustrated by its use in all stadiums and training facilities during the 2022 FIFA World Cup in Qatar.

Although there is some controversy regarding the origin of *P. vaginatum*, some authors agree that the species is native to South America and that it subsequently spread worldwide [3,4]. The species is predominantly diploid (2n = 2x = 20) and cross-pollinated due to self-incompatibility [5,6]. Vegetative propagation is the predominant method used for commercial production, although the species can also reproduce sexually. Genetic improvement has been achieved primarily through ecotype selection and intraspecific hybridization [1,7]. Seeded cultivars are scarce and correspond to improved populations obtained through recurrent phenotypic selection.

The possibility of hybridization between *P. vaginatum* and other diploid *Paspalum* species has been attempted with reduced or null success [8,9]. Nevertheless, most *Paspalum* species are polyploids and reproduce by apomixis [10], which may provide an alternative pathway to overcome hybrid sterility and facilitate the incorporation of genetic variation from related species. One example is *P. distichum* L., which is part of the Disticha group together with *P. vaginatum*, since both species share most morphological characteristics [3,4,11] and genetic composition [12]. *P. distichum* is polyploid, mainly tetraploid, and reproduces by apomixis [10]. There are also other species with turf attributes, such as *P. notatum* Flüggé and *P. conjugatum* P.J. Bergius, which are also predominantly tetraploid and apomictic [6].

Hybridization at the tetraploid level has been successfully accomplished in several *Paspalum* species through chromosome doubling of diploid germplasm, followed by crosses using the induced sexual tetraploid genotypes as female parents and apomictic germplasm as male parents [10]. This strategy has been successful because most induced tetraploids reproduce sexually, while meiosis proceeds normally in the male organs of apomictic parents. Hybridization at the tetraploid level between *P. vaginatum* and other *Paspalum* species would make it possible to explore interspecific relationships and develop new breeding strategies based on previously inaccessible sources of genetic variation. Furthermore, because a proportion of the progeny from crosses between sexual and apomictic parents inherits apomixis, and because apomixis can circumvent hybrid sterility, the apomictic progeny may be able to produce seeds regardless of the genetic distance between the species involved in the cross.

Induced polyploidization may result in a wide range of phenotypic and reproductive responses. In addition to affecting reproductive mode and fertility [13,14], chromosome doubling is often associated with the gigas effect, characterized by increased organ size and other morphological changes [15,16,17]. These effects highlight the importance of evaluating the impact of chromosome doubling on both phenotypic and reproductive traits. Schwartz et al. [18] attempted to induce polyploidy in *P. vaginatum* by treating seeds of the diploid cultivar ‘SeaStar’ with colchicine. Although they obtained a triploid plant exhibiting narrow leaves and darker green coloration, no artificially induced tetraploid genotypes were identified. Consequently, the phenotypic and reproductive effects of chromosome doubling leading to tetraploidy in *P. vaginatum* remain largely unknown. In particular, it is unclear whether induced tetraploids can be obtained and whether chromosome doubling affects their morphology, fertility, and reproductive mode.

The objectives of this research were to generate a tetraploid genotype of *P. vaginatum* by doubling the chromosome number of a diploid genotype, characterize and compare the phenotype of the induced tetraploid and the diploid, and evaluate the effect of chromosome duplication on critical reproductive characteristics.

This induced tetraploid genotype represents a valuable tool for future interspecific hybridization with other tetraploid *Paspalum* species, offering new opportunities to broaden the genetic base of *P. vaginatum* and to develop improved turf cultivars with enhanced stress tolerance and turf quality.

## 2. Results

### 2.1. Generation of an Induced Tetraploid Plant

**Figure 1:**
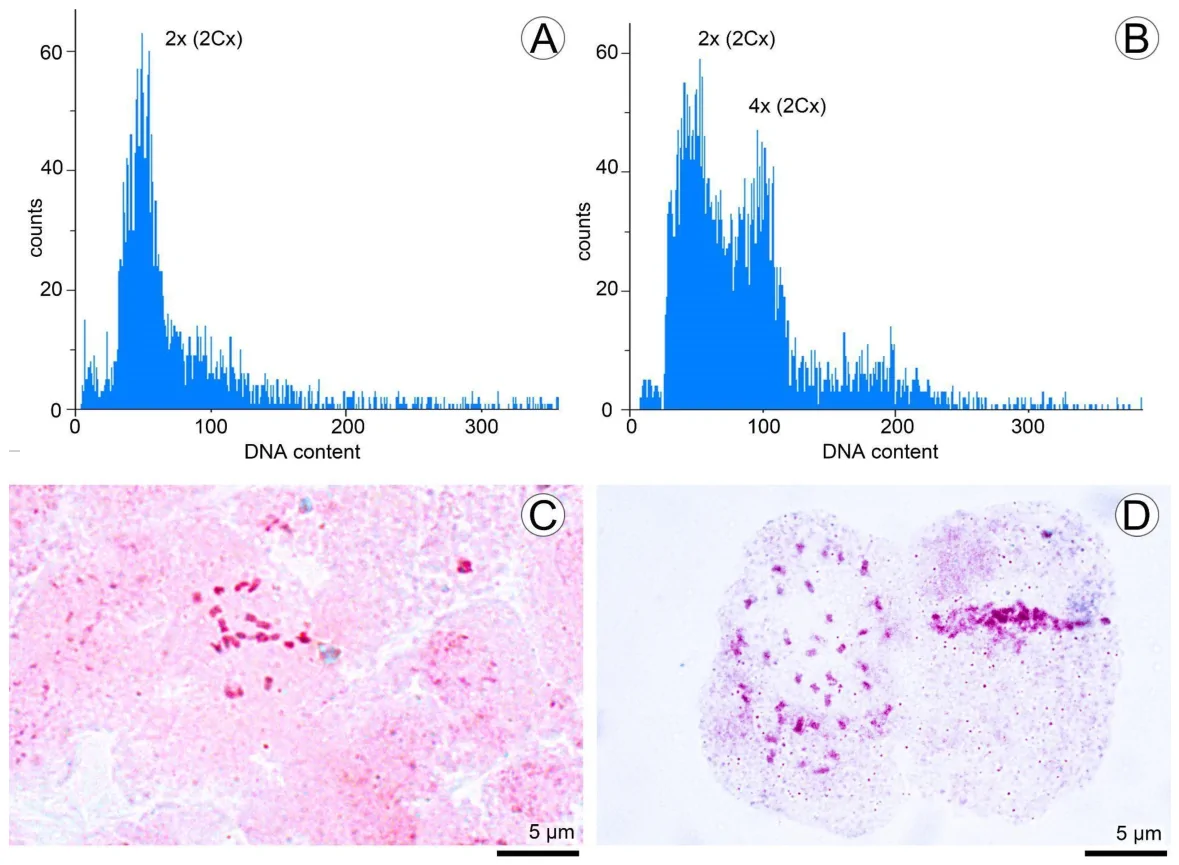
Flow cytometry analysis and nuclear staining in *Paspalum vaginatum* cytotypes. (**A**,**B**) DNA content histograms obtained by flow cytometry using nuclei isolated from young leaf tissue. (**A**) Diploid cytotype (2×), showing a single fluorescence peak. (**B**) Mixed sample obtained by co-chopping diploid and tetraploid tissues showing peaks corresponding to the diploid (2n = 2x = 20) and tetraploid (2n = 4x = 40) cytotypes. (**C**,**D**) Mitotic metaphase chromosomes obtained from root-tip meristematic tissue. (**C**) Diploid cytotype (2×). (**D**) Tetraploid cytotype (4×).

Caryopses and seedlings of diploid *P. vaginatum* were treated with 0.2% colchicine for 24 and 48 h to induce chromosome duplication (Table 1). The ploidy level of all surviving colchicine-treated plants was determined by both flow cytometry and chromosome counting. When caryopses were treated for 24 h, 96 plants germinated (13.7%), and a single one survived (1.0% of germinated plants), which was diploid (Figure 1A,C). When caryopses were treated for 48 h, 71 plants germinated (10.1%), but none survived. Treatment of 460 seedlings for 24 h resulted in 19 surviving plants (4.1%); 18 were diploid, and one was tetraploid (Figure 1B,D). Furthermore, three diploid plants (7.5%) survived following the 48 h treatment of 40 seedlings. Untreated (T0 = 0 h) caryopses and seedlings were included as controls to assess baseline germination and survival. Of 200 control caryopses, 92 germinated (46.0%), and 90 survived (97.8% of germinated plants); of 50 control seedlings, 45 survived (90.0%). Ploidy of surviving control plants was assessed by flow cytometry only, and all were confirmed as diploid. No mixoploid or tetraploid individuals were detected among untreated caryopses or seedlings, indicating that the tetraploid plant obtained after the 24 h colchicine treatment of seedlings did not arise from spontaneous chromosome doubling.

### 2.2. Phenotypic Characterization

**Figure 2:**
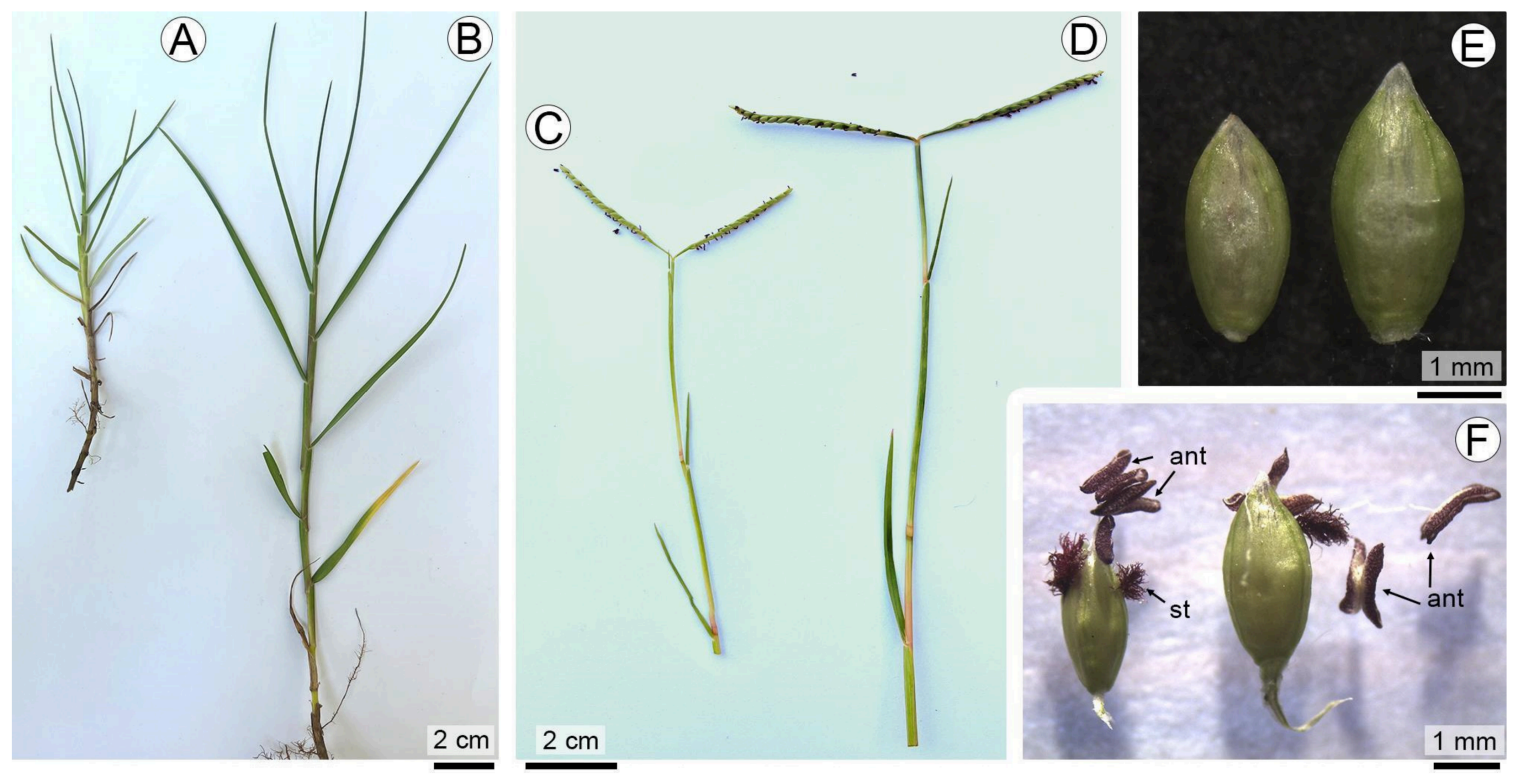
Vegetative and reproductive morphology of *Paspalum vaginatum*; (**A**,**C**) diploid cytotype (2×) and (**B**,**D**) tetraploid cytotype (4×). (**A**,**B**) Habit of the plant showing vegetative shoots and culms. (**C**,**D**) Flowering culms with the characteristic digitate inflorescence composed of two racemes. (**E**) Spikelets before the anthesis of the diploid (2×, **left**) and tetraploid (4×, **right**) cytotypes. (**F**) Spikelets at anthesis of the diploid (2×, **left**) and tetraploid (4×, **right**) cytotypes, showing the exposed stigmas (st) and anthers (ant).

A group of morphological, anatomical and physiological characteristics were measured in the diploid and the induced tetraploid genotype (Table 2). Stolon’s apex to fourth node length (Table 2; Figure 2A,B), internode length (Table 2; Figure 2A,B) and width, node width, leaf blade length (Table 2; Figure 2A,B), width and thickness, upper and lower raceme length (Table 2; Figure 2C,D), spikelet length and width were significantly greater in the tetraploid genotype in comparison to the diploid one (Table 2; Figure 2E,F). The most pronounced changes were observed in leaf blade length (227.1%), leaf blade width (69.6%), internode length (58.4%), leaf thickness (46.4%), lower raceme length (44.6%), and stomatal length (32.2%). Both genotypes had eight lived leaves per tiller (Table 2; Figure 2A,B).

**Figure 3:**
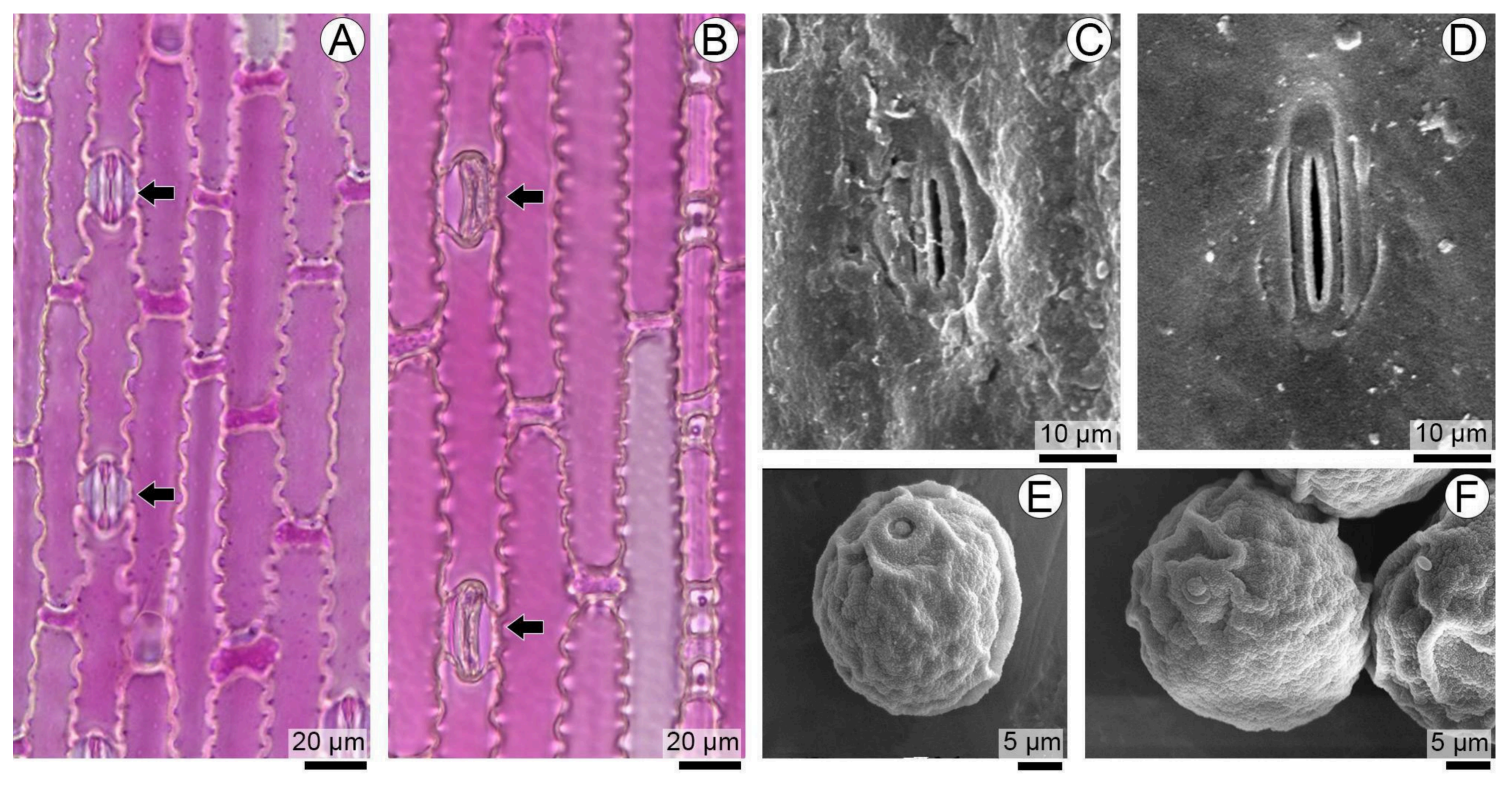
Leaf micromorphology and pollen morphology of *Paspalum vaginatum*. (**A**,**C**,**E**): Diploid cytotype (2×). (**B**,**D**,**F**): Tetraploid cytotype (4×). (**A**,**B**) Cleared leaf blades showing stomata (arrows) on the abaxial epidermis. (**C**,**D**) Scanning electron microscopy (SEM) images of stomata on the leaf epidermis. (**E**,**F**) SEM images of pollen grains showing general morphology and exine ornamentation.

The stomatal length and pollen equatorial diameter were also higher for the tetraploid than the diploid (Figure 3).

**Figure 4:**
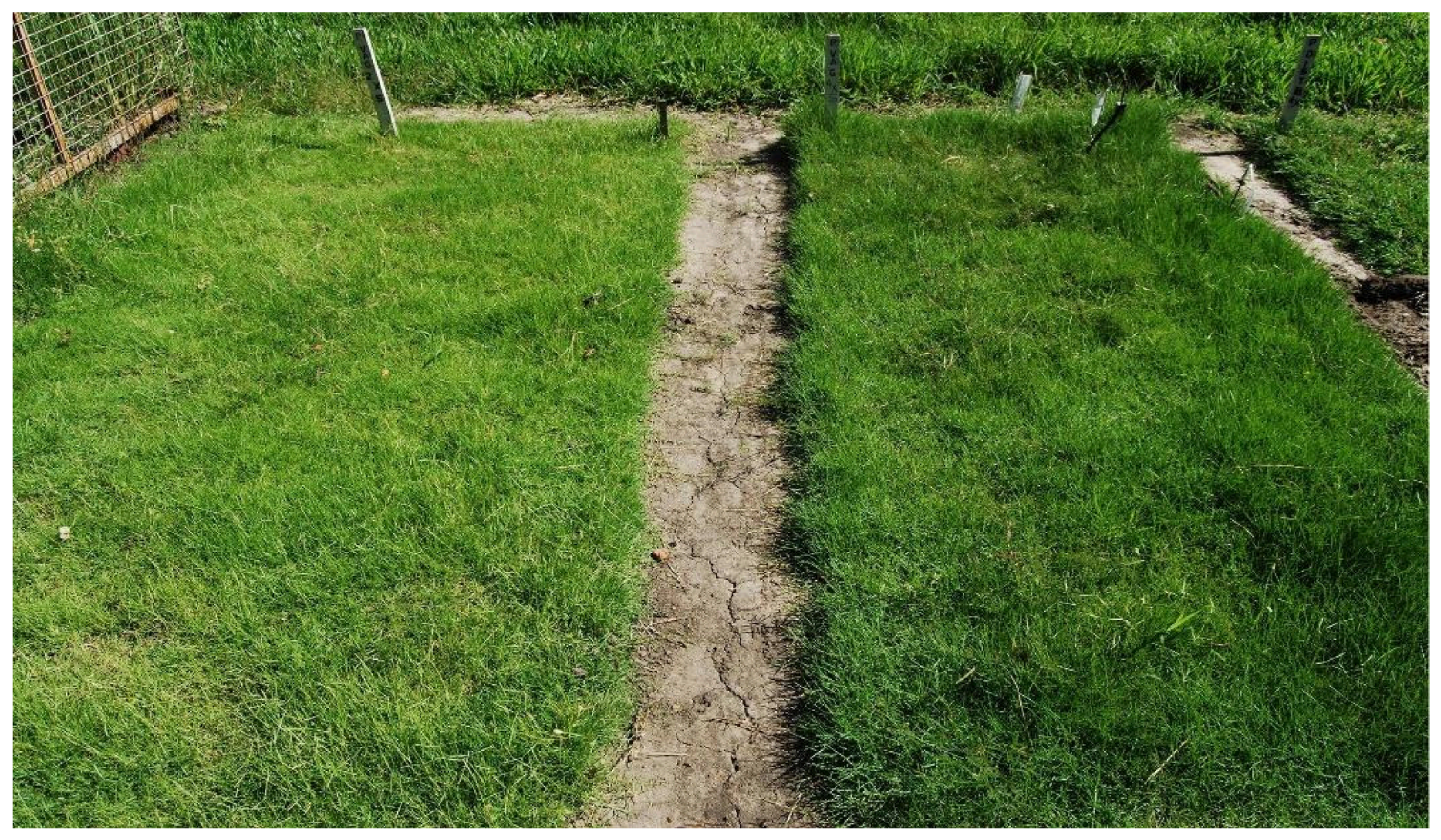
Color comparison between *Paspalum vaginatum* cytotypes. **Left**: diploid cytotype (2×); **right**: induced tetraploid cytotype (4×).

Additionally, the tetraploid genotype exhibited a darker color in comparison with the diploid, and that resulted in a significantly higher NDVI value (Figure 4).

### 2.3. Reproductive Characterization

**Figure 5:**
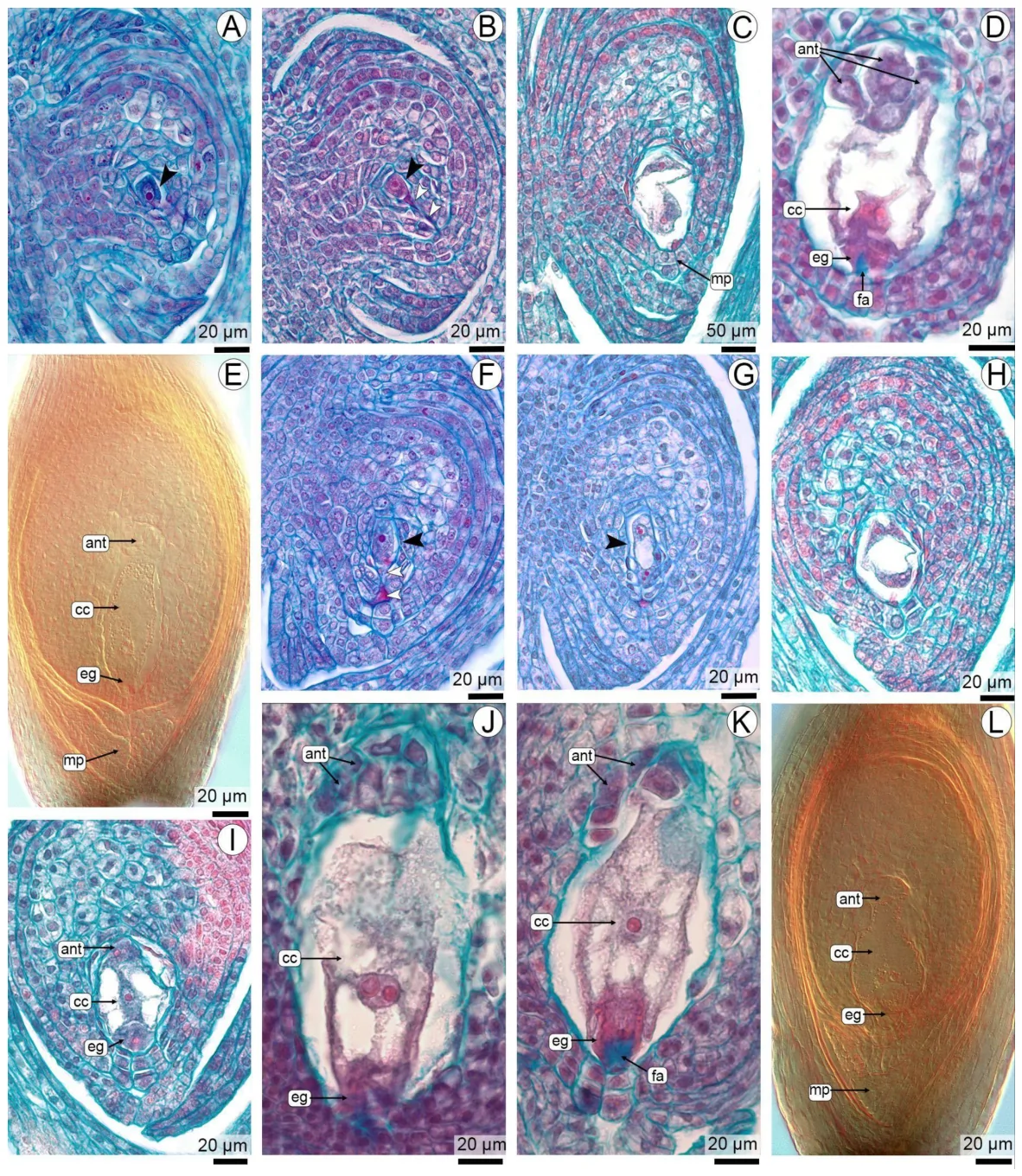
Megasporogenesis and embryo sac development in *Paspalum vaginatum*. (**A**–**E**) 2×. (**A**) Ovule with the megaspore mother cell (black arrowhead). (**B**) Functional chalazal megaspore (black arrowhead) and degenerated micropylar megaspores (white arrowheads). (**C**) Developing embryo sac at the 4-nucleate stage (only two nuclei are visible in this plane of section). (**D**) Mature embryo sac showing the egg apparatus, central cell, and mass of antipodals. (**E**) Cleared mature ovule showing the overall organization of the female gametophyte. (**F**–**L**) 4×. (**F**) Functional chalazal megaspore (black arrowhead) and degenerated micropylar megaspores (white arrowheads). (**G**) Developing embryo sac at the 2-nucleate stage (black arrowhead). (**H**) Developing embryo sac at the 4-nucleate stage. (**I**) Young embryo sac at 8-nucleate stage. (**J**) Mature embryo sac showing part of the egg apparatus, proliferated antipodals, and one nucleus of the central cell. (**K**) Mature embryo sac, showing the second nucleus of the central cell and the synergids with a well-developed filiform apparatus. (**L**) Cleared mature ovule showing the fully developed gametophyte. Abbreviations: ant, antipodals; cc, central cell; eg, egg apparatus; fa, filiform apparatus; mp, micropylar pole.

Megasporogenesis and megagametogenesis followed the same developmental sequence in the diploid (Figure 5A–E) and tetraploid cytotypes (Figure 5F–L). In both cytotypes, a single megaspore mother cell differentiated within the nucellus and underwent meiosis, producing a linear tetrad of megaspores (Figure 5A,B). The three micropylar megaspores degenerated, whereas the chalazal megaspore remained functional (Figure 5B,F).

The functional chalazal megaspore enlarged and underwent successive mitotic divisions. Two-, four-, and eight-nucleate stages were observed during embryo sac development (Figure 5C,G–I). At maturity, the female gametophyte comprised an egg apparatus consisting of one egg cell and two synergids, a large central cell containing the polar nuclei, and a multicellular antipodal cluster at the chalazal pole (Figure 5D,E,H,J–L). The synergids displayed a conspicuous filiform apparatus (Figure 5D,J,K).

All ovules examined showed a single embryo sac derived from the chalazal megaspore. At no stage were structural or developmental features indicative of apomixis detected. In particular, there was no evidence of aposporous embryo sac formation, multiple embryo sacs of independent origin within a single ovule, or deviations from the meiotic origin of the functional megaspore.

### 2.4. Seed Fertility

Neither the diploid nor the induced tetraploid genotypes set seed under self-pollination (Table 3). In contrast, the 2× genotype achieved a seed set of 23.3% under open-pollination. The induced tetraploid genotype also produced seeds under open-pollination, although seed set was much lower, reaching only 0.9% of the collected spikelets.

## 3. Discussion

A single induced tetraploid plant of *P. vaginatum* was obtained by treating seedlings with 0.2% colchicine for 24 h, with an efficiency of 0.2% (1 duplicate/460 treated seedlings). Chromosome doubling induced by colchicine has also been reported for six other diploid *Paspalum* species, namely *P. notatum* [14,19,20], *P. simplex* Morong [21], *P. hexastachyum* Parodi [13], *P. plicatulum* Michx. [15], *P. rufum* Nees ex Steud. [22], and *P. chaseanum* Parodi [16]. In contrast, Schwartz et al. [18] obtained only a triploid plant when attempting to double the chromosome number of *P. vaginatum*. Therefore, to our knowledge, this is the first report of successful chromosome doubling in *P. vaginatum*. The low efficiency observed in our study may explain why chromosome duplication has not previously been reported in this economically important species.

Chromosome doubling in *P. vaginatum* had a clear effect on plant morphology and anatomy, as most of the evaluated characters increased in size. This phenomenon, known as the gigas effect [23], has been reported in several plant species. It also appears to be a general pattern in *Paspalum*, as Sartor et al. [15], Quesenberry et al. [20], and Novo et al. [16] reported increases in organ size following the polyploidization of diploid species.

Polyploidization also resulted in a darker green coloration in *P. vaginatum*, a response that was likewise observed in triploid plants by Schwartz et al. [18]. The increases in organ size and changes in plant coloration may represent useful sources of phenotypic variation for cultivar development. High vigor in the tetraploid *P. vaginatum* genotype was an unexpected finding, as chromosome-doubled *Paspalum* genotypes generally do not exhibit this characteristic. In contrast, wider tillers with longer internodes, wider or thicker leaves, and larger reproductive structures may negatively affect turfgrass quality and performance.

Megasporogenesis and megagametogenesis followed the same developmental sequence in the diploid and induced tetraploid cytotypes. In both cytotypes, the embryo sac originated from the chalazal product of meiosis and developed into a Polygonum-type female gametophyte, with a multicellular antipodal cluster consistent with the variation commonly reported in *Paspalum* [24]. These observations indicate that chromosome doubling did not produce detectable alterations in the sexual pathway of female gametophyte development. The conspicuous proliferation of the antipodal cells was also observed at both ploidy levels, suggesting that this feature was retained after chromosome duplication. Some chromosome-doubled *Paspalum* genotypes have been reported to exhibit low levels of apomictic expressivity [14] as a consequence of increased ploidy; however, this phenomenon was not observed in the present study. Additionally, neither the diploid nor the induced tetraploid set seed under self-pollination, and both produced only low seed set under open-pollination conditions. These results indicate that the induced tetraploid is cross-pollinated, as previously reported for the diploid cytotype [6].

The low seed set observed in the induced tetraploid may be related to the absence of cross-compatible germplasm in its vicinity. Therefore, further research is needed to determine the cross-compatibility of the induced tetraploid with other species, particularly with accessions of *P. distichum*, the only other species in the Disticha group together with *P. vaginatum* [10].

**Figure 6:**
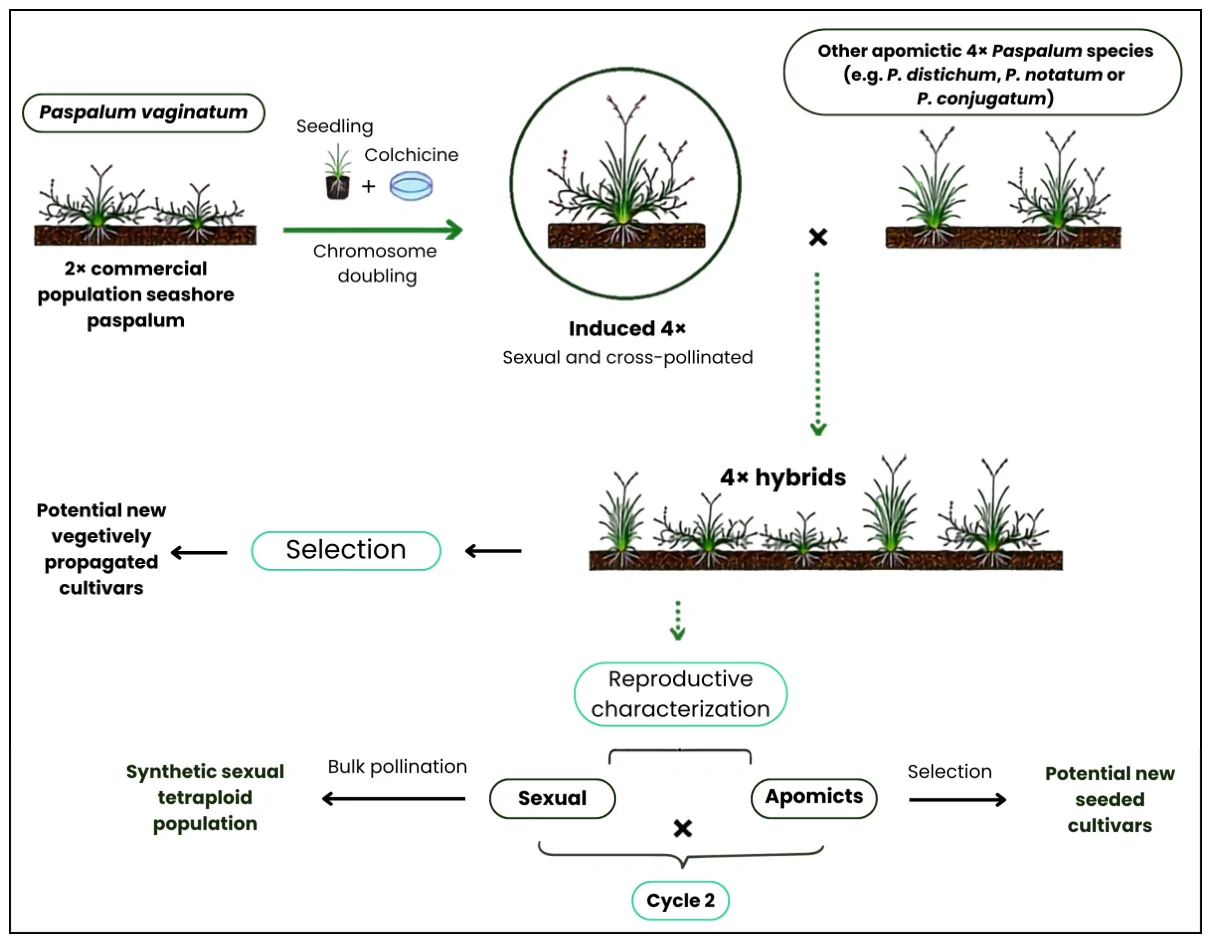
Proposed breeding scheme for *P. vaginatum* at the tetraploid level.

The development of a sexual, cross-pollinated tetraploid genotype of *P. vaginatum* opens entirely new opportunities for breeding. A novel breeding scheme is proposed in Figure 6, integrating the potential use of interspecific hybridization and the manipulation of apomixis. The induced tetraploid genotype may be used as a bridge parent (female parent) in crosses with accessions of *P. distichum* or other turf-type *Paspalum* species. The resulting segregating progeny could enter directly into a selection program aimed at identifying superior genotypes for vegetative propagation. The progeny is also expected to segregate for mode of reproduction, as reported for other *Paspalum* species [10]. The sexual progeny can be improved through different forms of recurrent selection, as described by Marcón et al. [25], whereas the apomictic progeny can be exploited for the selection of seeded cultivars. Thus, the main advantage of breeding the tetraploid germplasm compared with the diploid germplasm is the possibility of fixing superior hybrids through apomixis while enabling the introgression of genetic diversity from other *Paspalum* species with turf potential.

In conclusion, a tetraploid *P. vaginatum* genotype was successfully generated through chromosome doubling. The increase in ploidy resulted in phenotypic changes, primarily increases in cell and organ size, which may be useful for breeding. The chromosome-doubled genotype remained sexual and cross-pollinated and may serve as a bridge parent to introgress genetic variation from cross-compatible apomictic species.

## 4. Materials and Methods

### 4.1. Plant Material

A breeding population of *P. vaginatum* provided by the private company Zinma Seeds was used as the source material. Species identification and ploidy level were confirmed prior to the experiment. The induced tetraploid genotype used in this study was obtained from this diploid material as described below. Voucher specimens of both cytotypes were deposited in the Carmen L. Cristóbal Herbarium (CTES), Instituto de Botánica del Nordeste (IBONE), Corrientes, Argentina, under accession numbers CTES0076354 (diploid, 2×) and CTES0076355 (induced tetraploid, 4×).

### 4.2. Chromosome Duplication Treatments and Ploidy Determination

Four colchicine-based treatments were applied to induce chromosome duplication. Caryopses and seedlings were exposed to 0.2% colchicine for 24 or 48 h [15]. A control treatment (T0) was included, consisting of untreated plant material without colchicine exposure. Each treatment was conducted in independent replicates: caryopses were treated in three independent replicates for the 24 h treatment and three independent replicates for the 48 h treatment, while seedlings were treated in two independent replicates for the 24 h treatment and two independent replicates for the 48 h treatment. An untreated control (T0) was run in parallel with every replicate. Table 1 reports the pooled totals across replicates for each treatment. This concentration was selected based on previous studies reporting successful induction of polyploidy in *Paspalum* species [16,26]. After treatment, the plant material was thoroughly washed, and the caryopses were sown in trays containing sterile soil and maintained under greenhouse conditions. Germination was assessed 10 days after sowing. At the three-leaf stage, the seedlings were transplanted into pots and grown in the greenhouse until ploidy determination.

Ploidy level was estimated using the flow cytometry technique [27]. The fluorescence intensity of DAPI-stained nuclei was analyzed with a Sysmex CyFlow Space (Sysmex, Münster, Germany) flow cytometer. The ploidy level of each individual was determined using samples of fresh leaf tissue following the Sysmex recommendations of the Sysmex kit CyStain UV Precise P 05-5002 manual. In addition, chromosome number was confirmed through chromosome counts of root apical meristem cells [28].

### 4.3. Phenotypic Characterization

The diploid population and the induced tetraploid genotype were planted in the field in September 2024 in 5 m^2^ plots. Once the plots were completely covered, a group of morphological, anatomical and ecophysiological traits were measured to characterize the phenotype of the diploid and tetraploid germplasm. The length from the stolon apex to the fourth node, internode length and width, node width, leaf blade length and width, number of leaves per tiller, upper and lower raceme length were directly measured in the field using a caliper. Six tillers per plot were selected at equidistant points to measure these traits. Leaf-related traits were measured in the leaf adjacent to the inflorescence. The spikelet length and width were measured in the lab with a caliber. Leaf thickness, stomatal length, and pollen grain equatorial diameter were determined using Leica DM LB2 light microscope (LM, Leica Microsystems GmbH, Wetzlar, Germany) and ZEISS EVO 15 scanning electron microscopy (SEM, Carl Zeiss Microscopy GmbH, Jena, Germany), as appropriate. The sampling and procedures used for each microscopy technique are described in the following section. The Normalized Difference Vegetation Index (NDVI) was determined using a Greenseeker (Trimble, Handheld Crop Sensor, Westminster, CO, USA). The GreenSeeker was positioned at a height of 80 cm above the canopy for each measurement. Five stationary measurements were taken at equidistant points within each plot on 5 January at noon. The data was statistically analyzed by analysis of variance (ANOVA) using the statistical program InfoStat 2017 [29]. Fisher’s Least Significant Difference (LSD) test was used as the mean separation procedure for comparisons between the diploid and tetraploid germplasm.

### 4.4. Microscopy Analyses

Stereomicroscopy: Spikelets from the diploid and induced tetraploid cytotypes were examined using a Leica MZ6 stereomicroscope equipped with a Flexacam C1 digital imaging system (Leica Microsystems, Wetzlar, Germany).

Light Microscopy: For each cytotype, approximately 50 spikelets representing different developmental stages and 10 fully expanded flag leaves were collected. Spikelets and leaf samples were fixed in FAA (5 mL formaldehyde, 5 mL glacial acetic acid, and 90 mL 70% ethanol), dehydrated through a histological dehydration series, and embedded in paraffin [30,31].

Because the spikelets were very small and required lateral positioning to obtain longitudinal sections through the ovule and embryo sac, the protocol was adapted by adding a trace amount of carmine to one of the butyl alcohol baths preceding paraffin infiltration. Only enough carmine was added to impart a faint red coloration to the specimens. This temporary pre-staining facilitated visualization and accurate orientation of the spikelets during embedding. The carmine was removed during slide processing and did not interfere with subsequent histological staining.

Serial longitudinal sections of the spikelets and transverse sections taken from the middle portion of fully expanded flag leaf blades were obtained at a thickness of 8–10 µm using a MICROM HM325 rotary microtome (Thermo Fisher Scientific, Walldorf, Germany). The sections were stained with Safranin–Astra Blue [32], dehydrated, cleared, and permanently mounted in synthetic Canada balsam.

For stomatal analysis, samples of fully expanded flag leaves were cleared in a 10% NaOH solution for 8 h at room temperature (25 °C), thoroughly rinsed with distilled water, stained with safranin, and mounted in glycerin for microscopic observation [30].

Observations and photomicrographs were obtained using a Leica DM LB2 light microscope (Leica Microsystems GmbH, Wetzlar, Germany) equipped with a Leica ICC50 HD digital camera (Leica Microsystems GmbH, Wetzlar, Germany).

Scanning Electron Microscopy (SEM): FAA-fixed spikelets and leaf samples were dehydrated through a graded acetone series, critical-point dried using CO_2_, and sputter-coated with gold–palladium [33]. Samples were examined using a ZEISS EVO 15 scanning electron microscope (Carl Zeiss Microscopy GmbH, Jena, Germany) operated at 20 kV at the Electron Microscopy Service of the Universidad Nacional del Nordeste, Corrientes, Argentina.

Differential Interference Contrast Microscopy: Inflorescences were fixed at anthesis in FAA. Pistils were dissected out and clarified using the method described by Zilli et al. [34]. A minimum of 100 pistils from each cytotype were observed using differential interference contrast microscopy and a Leica DM2500 microscope and photographed with a Leica EC3 camera (Leica Microsystems GmbH, Wetzlar, Germany).

Spikelet dimensions, leaf thickness, stomatal length, and pollen grain equatorial diameter were measured from digital images using Fiji software (version 2.17.0) [35], irrespective of the imaging technique used for image acquisition and according to the image-analysis procedures reported by Gonzalez [36].

### 4.5. Seed Fertility

Seed set was evaluated under both self- and open-pollination in diploid and tetraploid genotypes. A 5 m^2^ plot was established using the same open-pollinated diploid population employed for chromosome duplication. An additional 5 m^2^ plot was planted with the induced tetraploid genotype and surrounded by plots containing two tetraploid genotypes of *P. distichum* to provide a source of potential compatible pollen.

Self-pollination was achieved by enclosing individual inflorescences in glassine bags supported by stakes before anthesis. To facilitate pollen transfer within the bags, inflorescences were gently shaken daily during the flowering period. Approximately one month after anthesis, bagged inflorescences were harvested. Additional mature inflorescences were collected from unbagged plants to estimate seed set under open-pollination.

Harvested inflorescences were threshed, and florets containing caryopses were separated from empty florets using an air column separator. Seed set was calculated as the percentage of florets containing caryopses relative to the total number of pollinated florets.

## Figures and Tables

**Table 1:** Induction of a tetraploid (4×) plant from diploid (2×) caryopses and seedlings of *Paspalum vaginatum* by colchicine treatment (T). Values represent the sum of individuals from independent replicates per treatment: three replicates for each of the 24 h and 48 h treatments in caryopses and two replicates for each of the 24 h and 48 h treatments in seedlings; each replicate was run together with its own untreated control (T0).

Materials	Treatment	Treated Individuals (no.)	Germinated		Surviving Seedlings		Ploidy of Surviving Plants	
			(no.)	(%)	(no.)	(%)	2×	4×
Caryopses	T0 = 0 h	200	92	46.0	90	97.8	90	-
	T1 = 24 h	700	96	13.7	1	0.1	1	-
	T2 = 48 h	700	71	10.1	0	0.0	-	-
Seedlings	T0 = 0 h	50	-	-	45	90.0	45	-
	T1 = 24 h	460	-	-	19	4.1	18	1
	T2 = 48 h	40	-	-	3	7.5	3	-

**Table 2:** Morphological, anatomical, and physiological characteristics of diploid and tetraploid *Paspalum vaginatum*. Values are means (†). Means followed by the same letter within a row are not significantly different according to Fisher’s protected LSD test (*p* ≤ 0.05). * *p* ≤ 0.05; ** *p* ≤ 0.001; ns = not significant. NDVI = Normalized Difference Vegetation Index.

Characteristics	*P. vaginatum*				
	2×		4×		Significance
	Mean †		Mean †		
NDVI	0.65	b	0.74	a	**
Apex to 4th node length (cm)	21.84	b	26.55	a	*
Internode length (cm)	7.53	b	11.93	a	**
Node width (cm)	2.36	b	3.06	a	**
Internode width (cm)	2.02	b	2.66	a	**
Leaves per tiller (n°)	8.00		8.00		ns
Leaf blade length (cm)	7.00	b	22.90	a	**
Leaf blade width (mm)	0.92	b	1.56	a	**
Leaf thickness (µm)	94.82	b	138.86	a	**
Stomatal length (µm)	27.99	b	37.00	a	**
Lower raceme length (cm)	30.95	b	44.75	a	**
Upper raceme length (cm)	32.45	b	41.90	a	**
Spikelet length (mm)	0.25	b	0.29	a	**
Spikelet width (mm)	0.11	b	0.14	a	**
Pollen equatorial diameter (µm)	25.23	b	27.84	a	**

**Table 3:** Proportion of spikelets that formed caryopses following self- and open-pollination in diploid and induced tetraploid plants of *Paspalum vaginatum*.

Cytotype	Self-Pollination		Open-Pollination	
	Mature Spikelets Analyzed (no.)	Seed Set (%)	Mature Spikelets Analyzed (no.)	Seed Set (%)
*P. vaginatum*				
2×	972	0.0	20,404	23.3
4×	596	0.0	13,039	0.9

## Data Availability

All the data presented in this study are available in this article.
